# Draft Genome Sequence of *Parabacteroides goldsteinii* with Putative Novel Metallo-β-Lactamases Isolated from a Blood Culture from a Human Patient

**DOI:** 10.1128/genomeA.00937-15

**Published:** 2015-08-20

**Authors:** Thøger Jensen Krogh, Charlotte Nielsen Agergaard, Jakob Møller-Jensen, Ulrik Stenz Justesen

**Affiliations:** a Department of Biochemistry and Molecular Biology, University of Southern Denmark, Odense, Denmark; b Department of Clinical Microbiology, Odense University Hospital, Odense, Denmark

## Abstract

*Parabacteroides goldsteinii* was isolated from a blood culture. Genomic DNA was sequenced using a MiSeq sequencer and assembled using the SPAdes genome assembler. The draft genome sequence was 6,851,868 bp, spanning 282 contigs of 5,253 coding sequences, 66 tRNAs, and 5 rRNAs. Several putative novel metallo-β-lactamases were discovered.

## GENOME ANNOUNCEMENT

*Parabacteroides goldsteinii* is an anaerobic, Gram-negative rod belonging to the microbiota of the human gut (1, 2).

Here, we report the draft genome sequence of a *P. goldsteinii* strain isolated from a blood culture of a human patient. Antimicrobial susceptibility testing with gradient MIC strips demonstrated resistance toward clindamycin (>256 mg/liter), piperacillin-tazobactam (>256 mg/liter), and meropenem (>32 mg/liter) but susceptibility toward metronidazole (0.38 mg/liter). A meropenem-EDTA double-ended gradient strip was positive, indicating the presence of a metallo-β-lactamase (3). In anaerobic bacteria, metallo-β-lactamases are usually demonstrated only in *Bacteroides fragilis* and encoded by the *cfiA* gene.

The genomic DNA of the *P. goldsteinii* strain was purified using the DNeasy blood and tissue kit (Qiagen), according to protocol.

Paired-end libraries with an insert size averaging 350 bp were generated using the Illumina Nextera DNA sample preparation kit. The DNA was sequenced via an Illumina MiSeq benchtop sequencer with 150-bp reads at a 30× theoretical coverage. Reads were merged using PEAR version 0.9.5 (4), and *de novo* genome assembly was done via SPAdes version 3.0 (5) available through Illumina BaseSpace.

The final assembly consisted of 282 contigs with an *N*_50_ of 60,312, totaling 6,851,868 bp, and with a GC content of 43.46%. For comparison, the three available sequenced *P. goldsteinii* strains listed on the NCBI genome database (strains dnLKV18, CL02T12C30, and DSM 19448) are 6.49 Mb to 7.09 Mb with a GC content of 43.30% to 43.50%.

Annotation was carried out via the NCBI Prokaryotic Genome Annotation Pipeline, identifying 5 rRNAs, 66 tRNAs, and 5,253 coding sequences (CDS), of which 2,268 of the latter code for purely hypothetical proteins with no obvious homology-inferred function. A search for resistance-associated genes via the ResFinder tool (version 2.1, accessed 2 June 2015) (settings, 30% ID, 20% length—lowest possible) (6) identified genes that accounted for clindamycin (*ermF*) and tetracycline (*tetX* and *tetQ*) resistance, but no genes that account for resistance toward piperacillin-tazobactam or meropenem.

Genome analysis led to the discovery of nine genes coding for putative novel β-lactamases, of which six are putative novel metallo-β-lactamases. All of the nine genes are located on different contigs with low to no internal primary sequence homology and low to no primary sequence homology for any of the β-lactamases in the ResFinder, Brenda, and NCBI databases (accessed 2 June 2015). Tertiary structure predictions via Phyre2 predict a general β-lactamase-like αββα fold for all nine CDS (7, 8). These findings may represent several new subclasses of β-lactamases.

### Nucleotide sequence accession numbers.

This whole-genome shotgun project has been deposited at DDBJ/EMBL/GenBank under the accession number LFJV00000000. The version described in this paper is version LFJV01000000.
